# The Correlation between the Frequency of Oral Lesions and the Amount of Smokeless Tobacco Usage in Patients Referred to Oral Medicine Department of Zahedan Dental School

**Published:** 2014-06

**Authors:** S. Lesan, T. Nosratzehi, MA. Ousia, F. Arbabikalati, E. Pourmardan

**Affiliations:** a Dept. of Oral Medicine Dentistry, Islamic Azad University of Medical Sciences, Tehran, Iran.; b Dept. of Oral Medicine Dentistry, Dental Research Center, Zahedan University of Medical Science, Zahedan, Iran.; c Dept. of Oral Medicine Dentistry, Genetics of Non Communicable Disease Research Center, Zahedan University of Medical Sciences, Zahedan, Iran.; d Dentist

**Keywords:** Smokeless tobacco, Snuff, Oral lesions

## Abstract

**Statement of Problem:** The increasing use of smokeless tobacco in the last 15 years has motivated researchers to evaluate its impact on its user’s health.

**Purpose: **The aim of this study was to determine the frequency of oral lesions related to smokeless tobacco and any possible correlation between the frequency of oral lesions and the amount of usage in patients referred to Zahedan Dental School.

**Materials and Method:** A total of 90 (77 male and 13 female) cases, all snuff consumers, were surveyed in this cross sectional study which was accomplished by employing questionnaires. The questionnaire included demographic information, type and amount of smokeless tobacco used and the location where it was placed in the mouth. The completion of the questionnaires was followed by oral clinical examinations of patients. The location of any lesion found in soft tissue, was recorded in questionnaire.

**Results:** The mean age for men and women was 47.7 and 55.61, respectively. A total of 38 cases (29 males, 9 females) were found to have oral lesions. 26 patients were using the snuff one pack per day. The common site of lesions was lower buccal sulcus. From 38 lesions, 32 lesions were found at the site of snuff placement. Most of the lesions were degree 1 and white in color. After taking biopsy from 32 lesions, 26 cases were detected as hyperkeratotic and 6 cases as epithelial dysplasia.

**Conclusion:** This study showed that use of snuff is very common in Zahedan and usage of this material can produce oral lesions. There is not a significant correlation between the frequency of oral lesions and the amount of usage.

## Introduction 

Tobacco use causes about 5000 deaths annually in Iran [1]. Every year, five million people die of tobacco-related diseases in the world. Smokeless tobacco consumption, whether chewing tobacco or snuff, has become a common finding in many countries [2]. Increasing consumption of smokeless tobacco during the past fifteen years has motivated researchers to evaluate its impact on its user’s oral health and hygiene [3]. The role of dentists is imperative in screening these cases to prevent the subsequent oral cancer and premalignant lesions, however, some dentists are unaware of the prevalence of this problem in their patients [4].

A dentist is often the first person who performs the oral examination; hence, having a key position in early diagnosis of the lesions [5]. Oral cancer and various oral mucosal lesions are associated with tobacco consumption. This acknowledges the significant role of public education in constraining the use of such substances [4].

Smokeless tobacco is an important factor in developing the oral carcinoma. The habit of chewing tobacco causes pouches in the contact area following the development of detectable mucosal while lesions, which are called snuff keratosis or tobacco pouch. Although all forms of tobacco may cause changes in the oral mucous membrane, snuff is more likely to cause oral lesions than chewing tobacco. The most affected area is the anterior mandible vestibule extending to posterior. Mucosal surface appears white, wrinkled and grainy [6].

Lesions are usually painless and asymptomatic and are diagnosed during a routine checkup. The duration of exposure to the substance is an important factor in the stimulation of mucosal irritation [6-7]. The possibility of malignant change is increased four times in chronic users of smokeless tobacco [7].

Winn studied the relation between tobacco usage and oral diseases and concluded that tobacco use is an important risk factor for oral cancer, mucosal lesions, periodontal diseases, delayed healing after periodontal treatment, gingival recession, and root and crown caries [8]. Available evidences declare that the risk of oral diseases is rising with tobacco consumption while withdrawal can reduce the risk. The high impact of tobacco use on oral diseases has also been proved [8].

Croucher et al. studied the oral health of Bangladeshi women who were tobacco users and surveyed its relation to oral pain [9]. They concluded that the reports of oral pain, at follow-up during a tobacco cessation program, relate to the occurrence of oral mucosa lesions at baseline. They recommended the appropriate dental care as a support for an effective tobacco use cessation [9].

Taybos studied the oral changes associated with tobacco use and demonstrated that tobacco use affects the surface epithelium, resulting in changes in the appearance of the tissues [10]. The changes may range from an increase in pigmentation to thickening of the epithelium (white lesion). Tobacco use can also irritate the minor salivary glands on the hard palate and directly increase a person’s risk for periodontal disease and oral cancer [10].

This study was carried out in Sistan- and- Baluchestan province, which is in vicinity of Afghanistan, Pakistan, and India. These products are illegally imported into the market in luxury packages. Thereby, smokeless tobacco use has become popular among the people of this province. This study aimed to investigate the prevalence of oral complications associated with non-smoking tobacco (pack year) in patients referred to Zahedan Dental School, Zahedan, Sistan- and-Baluchestan province. 

## Materials and Method

A total of 90 (77 male and 13 female) cases, all snuff consumers, were surveyed in this cross sectional study which was accomplished by filling questionnaires. The participants were smokeless tobacco users referred to the department of oral medicine, Zahedan Dental School in one year. The demographic data including age and gender were recorded in the questionnaire. The participants were assessed in terms of smokeless tobacco usage and subsequently the clinical examination of the oral cavity was performed. 

The data and information document of the participants was completed regarding the snuff and chewing-tobacco consumption. The Poison method has been opted as the sampling method.

The individuals who had used the chewing-tobacco for less than 6 months were excluded from the study. The amount of snuff and chewing-tobacco use was determined in data inventory form based on this equation: number of 30gram packs per day × number of days per year × number of years.

The patients were initially asked about their habits concerning the location where they place the snuff in their mouth. Then, two examiners inspected the soft tissue using mirrors and gauze. When a lesion was present, its location and color was registered in data inventory form. Then, the lesions were divided into three clinical grades: 

Grade 1: superficial and slight mucosal shrinkage in which mucosal color may range from normal to relatively white or gray and mucosal does not look thick; Grade 2: obvious color change to white, grey or relatively red in some cases, obvious shrinkage but no thickening; Grade 3: mucosa is markedly thickened with a relatively white or gray discoloration and deep grooves in thickened areas.  

In case of oral erosive or atrophic lesions, biopsy of the lesions was performed; the lesions were divided into two categories: hyperkeratosis and dysplasia. All the obtained data were evaluated and analyzed by employing Chi-square test using SPSS version 13. 

## Results

A total of 90 people were evaluated, 77 of which (85.6%) were male and 13 patients (14.4%) were female. The mean age was 47.4 and 55.61 years for men and women respectively. Among 90 examined individuals, 38 cases (42.2%) had oral lesions and 52 cases (57.8%) manifested no complications. In the group of 38 involved individuals, 29 patients (37.7%) were male and 9 patients (69.3%) were female. Of 52 patients who did not show oral complications, 48 cases (62.3%) were male and 4 cases (30.8%) were female. The Chi-squared test and Fisher's exact test showed that there was a significant difference (*p*= 0.034) between the two genders in terms of prevalence of oral complications following the use of pack year of 38 patients with a lesion, 17 patients (50%) had consumed 1501 to 5000 packets per year (Table 1).

**Table 1:** Comparison of the prevalence of oral lesions in terms of the consumption amount of pack year in patients consuming pack year referred to Zahedan Dental School

**Lesion frequency** ** Number of packets per year**	**With lesion**		**Without lesion**	
	**Number**	**Percent**	**Number**	**Percent**
Less than 500	5	23.8	16	76.2
5001-1500	8	40	12	60
1501-5000	17	50	17	50
5001-10000	3	37.5	5	62.5
More than 10000	5	71.4	2	28.6

Using the Chi-squared test, a comparison was made between the amount of consumption (number of packets per year) and the prevalence of oral lesions. The statistical results showed no difference between the amount of consumption and the prevalence of oral lesions (*p*= 0.176).

It was also found that 31 individuals put the substance in the mandibular labial vestibule (Table 2); the most affected place was the mandibular buccal vestibule (Table 3). Of 38 detected lesions; 63.16% were grade 1 and 68.43% were white in color (tables 4 and 5). After performing the biopsy on 32 lesions, (72.7%) of lesions were found to be hyperkeratotic type and (27.3%) had epithelial dysplasia (Table 6).

**Table 2:** Frequency distribution of putting place of pack year in the mouth in patients consuming pack year referred to Zahedan Dental School

**Frequency** ** Putting place of pack year in the mouse**	**Number**	**Percent**
Mandibular labial vestibule	31	34.5
Mandibular buccal vestibule	29	32.2
Floor of the mouth	15	16.8
Mandibular labial and buccal vestibule	4	4.4
Mandibular buccal vestibule and floor of the mouth	3	3.3
Maxillary buccal vestibule	2	2.2
Maxillary buccal and labial vestibule	2	2.2
Maxillary labial vestibule	1	1.1
Edge of the tongue	1	1.1
Mandibular buccal vestibule and lower lip	1	1.1
Mandibular buccal vestibule and floor of the mouse	1	1.1
Gingiva and alveolar ridge and commissure	-	-

**Table 3:** Frequency distribution of oral lesion location in patients consuming pack year referred to Zahedan Dental School

**Frequency** ** Location of oral lesion**	**Number**	**Percent**
Mandibular buccal vestibule	15	16.8
Mandibular labial vestibule	6	6.7
Floor of the mouth	4	4.4
Edge of the tongue	3	3.3
Mandibular labial and buccal vestibule	2	2.2
Maxillary buccal vestibule	1	1.1
Maxillary labial vestibule	1	1.1
Gingiva and alveolar ridge	1	1.1
Mandibular buccal vestibule, and gingiva and alveolar ridge	1	1.1
Mandibular buccal vestibule and tongue	1	1.1
Maxillary labial and buccal vestibule	1	1.1
Mandibular buccal and labial vestibule and tongue, Gingiva and alveolar ridge	1	1.1
Mandibular buccal and labial vestibule and tongue, Gingiva and alveolar ridge, lower lip and commissure	1	1.1

**Table 4:** Frequency distribution of lesion clinical grade in patients consuming pack year

**Frequency** ** Clinical grade of lesion**	**Number**	**Percent**
Grade 1	24	63.16
Grade 2	6	15.78
Grade 3	8	21.06
Total	38	100

**Table 5:** Frequency distribution of lesion color in patients consuming pack year

**Frequency** ** Color of lesion **	**Number**	**Percent**
White	26	68.43
Red	3	7.89
Red and white	9	23.68
Total	38	100

**Table 6:** Frequency distribution of oral lesion type after performing the biopsy in patients consuming pack year referred to Zahedan Dental School

**Frequency** ** Type of lesion**	**Number**	**Percent**
Hyperkeratosis	26	72.7
Epithelial dysplasia	6	27.3
Total	32	100

## Discussion

The current research revealed that using snuff highly occurs in Zahedan city and can develop oral lesions; even though no significant relationship was established between the frequency of oral lesion and the amount of snuff consumption. The number of male snuff users was more than of female consumers. These findings are consistent with Wolfe and Kaugars claiming that snuff use is more prevalence amongst males [11-12].

The study showed that female participants were more affected by snuff usage. This result was in accordance with Wray and Guirt study [13], and in contrast to Kaugars et al. study [11]. This different result might have been because female snuff users in Zahedan were using the substances for longer periods.

The majority of snuff users in the current were between 35-54 years old. This finding is in agreement with Wray and Guirt study [13] that reported the middle-aged are the biggest group of snuff users. The findings of this study showed that there was no significant difference between various age groups with respect to oral lesion involvement. These results are in contrast to the findings of Reichart and Mohr [14] and Wray and Guirt studies [13]. This might be due to the wide range of age of participants in our study.

The findings of this study also showed that there was no significant correlation between the consumption rate and the prevalence of oral complication in the sample under investigation. These results do not correspond to the Wolfe and Carlos study [12] and Winn research [8]. It may be because of the small number of samples enrolled in the study and the popularity of snuff usage within the studied sample. The other reason could be due to the fact that despite high rates of snuff consumption; some people locate the substance in different areas of the mouth. This can be the reason that causes the oral complication not to be observed.

In the present study, the most common location for placing a snuff by the participants was the mandibular labial vestibule. The results are consistent with the findings of Ayo-Yusuf et al. enrolled in 2000 [15].

In the current study, the lesions were mostly seen in the mandibular buccal vestibule. It is in accordance with the study of Wray and Guirt [13] and the study of Kaugars et al. [11].

A significant correlation between the location of placing smokeless tobacco and the location of oral lesions were identified in this study (*p*= 0.0001) which is also in agreement with the findings of Kaugars et al. [11], Wray and Guirt [13] and Ayo-Yusuf et al. [15].

Most of the clinical lesions identified in this study were first-grade lesions and hyperkeratosis. These findings are consistent with the results obtained by Ayo-Yusuf et al. [15].

## Conclusion

The present research showed that using snuff highly occurs in Zahedan city and can develop oral lesions; although no significant relationship was found between the frequency of oral lesion and the amount of snuff use. 
